# Aberrantly activated Cox-2 and Wnt signaling interact to maintain cancer stem cells in glioblastoma

**DOI:** 10.18632/oncotarget.19283

**Published:** 2017-07-17

**Authors:** Megan Wu, Jennifer Guan, Chris Li, Simon Gunter, Labeeba Nusrat, Sheena Ng, Karan Dhand, Cindi Morshead, Albert Kim, Sunit Das

**Affiliations:** ^1^ Arthur and Sonia Labatt Brain Tumour Research Centre, Hospital for SickKids, University of Toronto, Toronto, Canada; ^2^ Department of Neurosurgery and Cell Biology, Washington University, St. Louis, MO, USA; ^3^ McLaughlin Centre for Molecular Medicine, University of Toronto, Toronto, Canada; ^4^ Division of Neurosurgery and Li Ka Shing Knowledge Institute, St. Michael’s Hospital, University of Toronto, Toronto, Canada

**Keywords:** glioblastoma, cancer stem cells, cyclooxygenase, prostaglandin E2, Wnt

## Abstract

Glioblastoma recurrence after aggressive therapy typically occurs within six months, and patients inevitably succumb to their disease. Tumor recurrence is driven by a subpopulation of cancer stem cells in glioblastoma (glioblastoma stem-like cells, GSCs), which exhibit resistance to cytotoxic therapies, compared to their non-stem-cell counterparts. Here, we show that the Cox-2 and Wnt signaling pathways are aberrantly activated in GSCs and interact to maintain the cancer stem cell identity. Cox-2 stimulates GSC self-renewal and proliferation through prostaglandin E2 (PGE2), which in turn activates the Wnt signaling pathway. Wnt signaling underlies PGE2-induced GSC self-renewal and independently directs GSC self-renewal and proliferation. Inhibition of PGE2 enhances the effect of temozolomide on GSCs, but affords only a modest survival advantage in a xenograft model in the setting of COX-independent Wnt activation. Our findings uncover an aberrant positive feedback interaction between the Cox-2/PGE2 and Wnt pathways that mediates the stem-like state in glioblastoma.

## Highlights

- Cox-2 and Wnt signaling pathways are aberrantly activated in GSCs
- Cox-2 stimulates GSC self-renewal and proliferation through prostaglandin E2 and activated Wnt
- Wnt signaling underlies PGE2-induced GSC self-renewal and independently directs GSC self-renewal and proliferation
- PGE2 inhibition enhances temozolomide cytotoxicity but fails to prevent tumor progression because of COX-independent Wnt activation

## INTRODUCTION

Despite advances in therapy, outcomes for patients with glioblastoma remain dismal. Disease recurrence after surgery, radiation and chemotherapy, typically occurs within six months, and patients inevitably succumb to disease progression at just over one year [1]. Emerging recognition of the cellular heterogeneity within glioblastoma has focused attention on a subpopulation of cancer stem cells (CSCs) in glioblastoma, called brain tumor-initiating cells or glioblastoma stem-like cells (GSCs) [2], that are thought to contribute to tumor recurrence following therapy. Compared to their non-stem-cell counterparts (differentiated glioma cells, DGCs), GSCs exhibit resistance to chemotherapy- and radiation-induced cell death [3–5], often through co-option of signaling pathways relevant to normal stem cell physiology [6–9]. Conceivably, these pathways could be therapeutic targets to disable CSC-mediated treatment resistance.

Cyclooxygenase-2 (Cox-2) is an inducible enzyme that plays a key role in the production of the bioactive lipid, prostaglandin E2 (PGE2). Cox-2 has been shown to play an important role in several human cancers, including glioblastoma. In prostate and colon cancer, elevated Cox-2 expression has been associated with increased angiogenesis, tumor invasion and promotion of tumor cell resistance to apoptosis [10–13]. Elevated Cox-2 levels have also been found to correlate with earlier recurrence and shorter survival in patients with gliomas [14].

In addition to its roles in angiogenesis, tumor invasion, and tumor cell survival, the Cox-2/PGE2 pathway may play a role in maintaining the cancer stem-like cell population and preserve self-renewal capacity in GBM. Cox-2 has been found to regulate glioma stem-like cell (GSC) proliferation [15, 16], and Cox-2-derived PGE2 promotes self-renewal and imparts radiation resistance in an RCAS-Tva GBM mouse model [17] and in multiple glioblastoma cell lines [18].

In the hematopoietic stem cell niche, Cox-2 regulates vertebrate HSC induction and engraftment directly through production of PGE2 [19], and indirectly through stabilization of β-catenin protein and activation of the Wnt pathway [20]. In the adult mouse brain, activation of the canonical Wnt signaling (Wnt/β-catenin pathway) is not seen under resting state conditions, but is expressed when progenitor cells are dividing symmetrically, that is, during subependymal zone regeneration, following stroke, or during neurosphere formation [21]. Aberrant activation of Wnt signaling in the subventricular zone results in symmetric expansion of the NSC pool. Interestingly, the activating effect of Wnt on embryonic stem cell self-renewal has been found to depend on its induction of Egr1 [22], which has also been implicated as a downstream target of the Cox-2 pathway [23].

Cancer stem cell systems have been described as hierarchies in which the normal mechanisms by which stem cells systems are maintained have been dysregulated to allow uninhibited cell expansion and growth [24]. Here, we show that Cox-2 is constitutively activated in human GSCs, but not in normal human neural stem cells (NSCs). Cox-2 signaling and production of PGE2 results in increased GSC proliferation and self-renewal in both GSCs and NSCs *in vitro*, while inhibition of Cox-2 in GSCs induces cell differentiation and loss of the cancer stem-like cell phenotype. PGE2 further promotes the GSC state through its activation of the Wnt/β-catenin pathway, which mediates PGE2-induced GSC self-renewal through its canonical pathway. Our findings uncover an aberrant interaction between the Cox-2/PGE2 and Wnt pathways that mediates the stem-like state in glioblastoma.

## RESULTS

### Cox-2 is expressed in human glioblastoma

**Figure 1:**
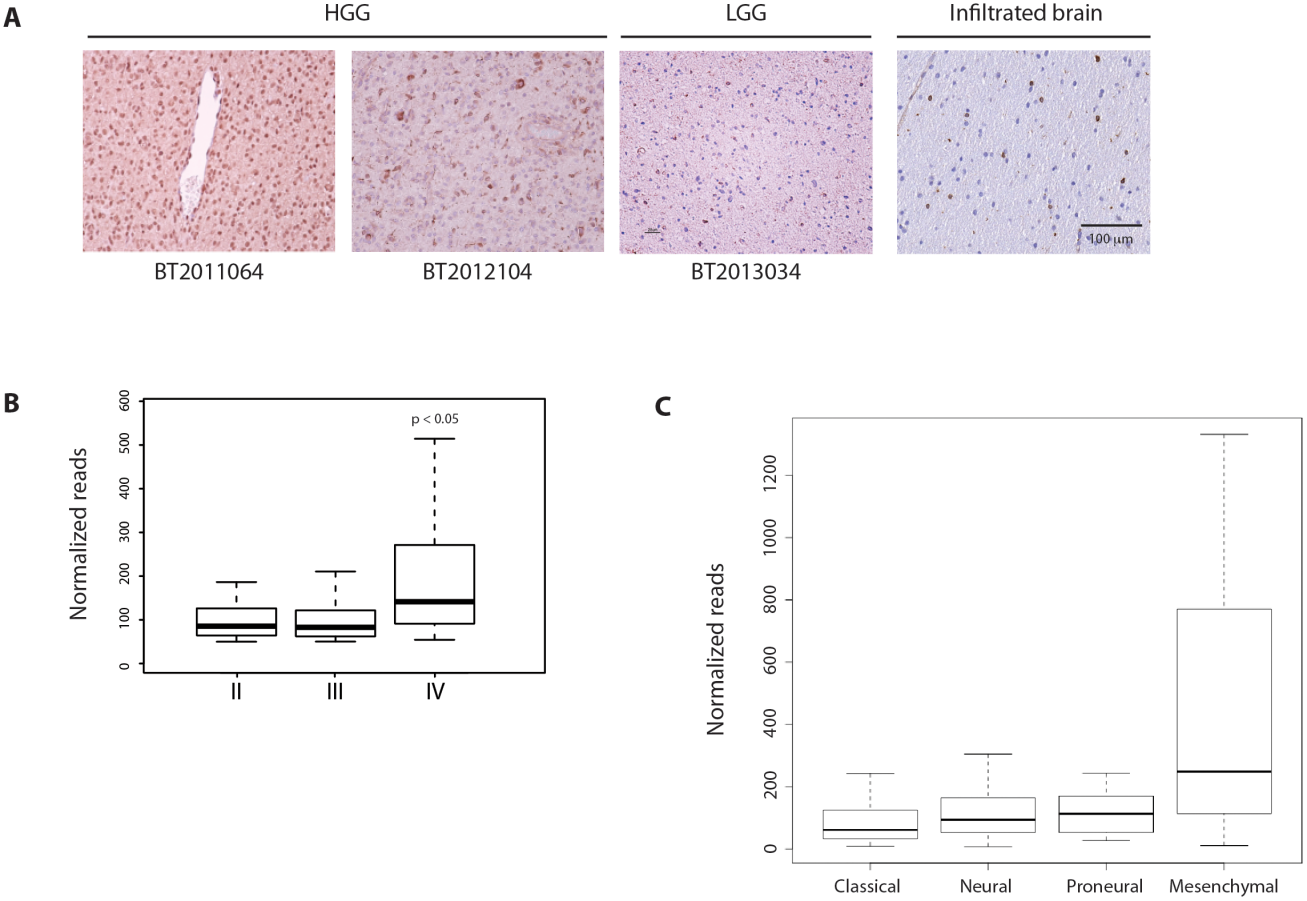
Cox-2 is expressed in human glioblastoma **(A)** Immunohistochemistry for Cox-2 shows abundant expression in high-grade glioma (BT2011064, BT2012104), with lower expression in low-grade glioma (BT2013034) and infiltrated brain. **(B, C)** Analysis of expression data from The Cancer Genome Atlas (TCGA). Cox-2 mRNA levels increase with increasing glioma grade and is enriched in the mesenchymal subgroup. Data are represented as mean ± SEM.

To examine Cox-2 expression in human glioma, we first performed immunohistochemistry for Cox-2 in low- and high-grade glioma surgical specimens. These studies showed Cox-2 expression in gliomas and in glioma cells within infiltrated brain (Figure 1A). We found no expression of Cox-2 within the normal brain hemisphere or within the neurogenic regions of the adult mouse subventricular zone or subgranular zone (Supplementary Figure 1). Examination of glioma expression data from The Cancer Genome Atlas (TCGA) demonstrated that Cox-2 expression increases with increasing tumor grade (Figure 1B) and is enriched in the mesenchymal subgroup (Figure 1C).

### Cox-2 is enriched in GSCs and promotes GSC proliferation through PGE2

**Figure 2:**
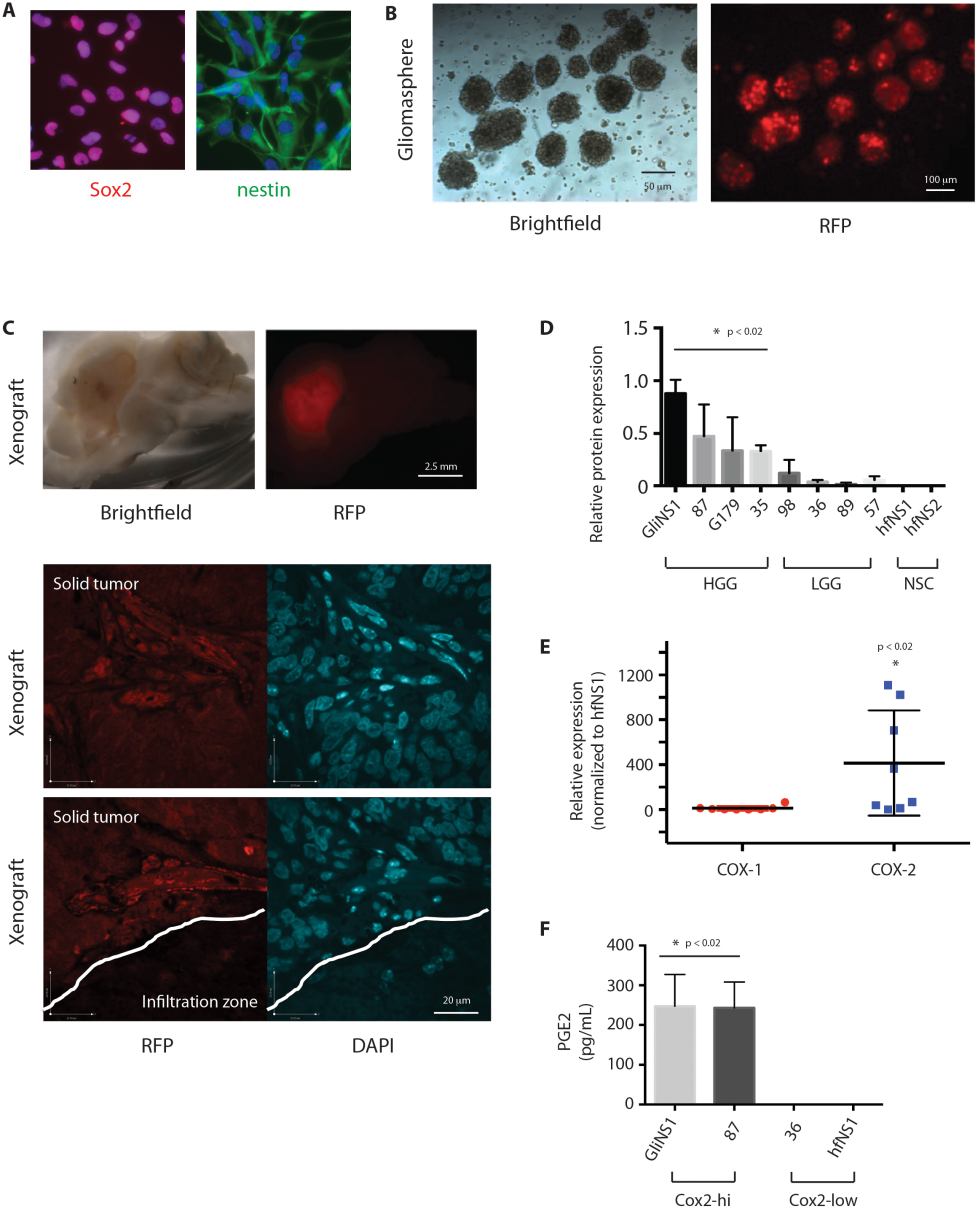
Cox-2 is enriched in GSCs and results in production of PGE2 **(A)** GSC lines were subjected to immunofluorescence with antibodies against Sox2 (red) or nestin (green), and Hoechst nuclear stain (blue). **(B)** RFP-expressing GSCs were grown as gliomaspheres and **(C)** injected into the right putamen of NOD-SCID mice. Animals were sacrificed after 2 months. Sectioned brains were subjected to Brightfield microscopy and immunofluorescent microscopy for RFP. **(D)** Relative quantification of COX-2 protein levels as determined by Western blot analysis of lysates from HGG-derived GSCs (GliNS1, 2012087, G179, 2014035), LGG-derived GSCs (2015098, 2014036, 2015089, 2014057), and human fetal NSCs (hfNS1, hfNS2). **(E)** Cox-1 and Cox-2 protein expression in GSCs (GliNS1, 2012087, G179, 2014035, 2015098, 2014036, 2015089, 2014057) compared to hfNSCs (hfNS1, hfNS2). **(F)** PGE2 production in GSCs with high (GliNS1, 2012087) or low (2015089, 2014057) basal Cox-2 expression. Data are represented as mean ± SEM.

As Cox-2 has been implicated as a mediator of the cancer stem cell phenotype in mouse glioblastoma, we sought to determine if it has an analogous role in the human disease. We generated low-passage RFP-expressing patient-derived GSC lines that express neural stem cell markers (Figure 2A), form self-renewing neurospheres (Figure 2B), and give rise to infiltrative brain tumors following orthotopic transplantation in immunocompromised mice [25] (Figure 2C). Western blot analysis utilizing GSCs and NSCs in culture confirmed that Cox-2 is basally expressed in GSCs, but not in human fetal NSCs (Figure 2D). Analogous to gross tumor expression data from TCGA, Cox-2 expression is higher in GSCs generated from high-grade gliomas than in GSCs generated from low-grade gliomas in culture. We found no evidence of basal Cox-1 expression in either GSCs or NSCs (Figure 2E). Prostaglandin E2 (PGE2) production was limited to glioma cells with high levels of basal Cox-2 expression (Figure 2F).

**Figure 3:**
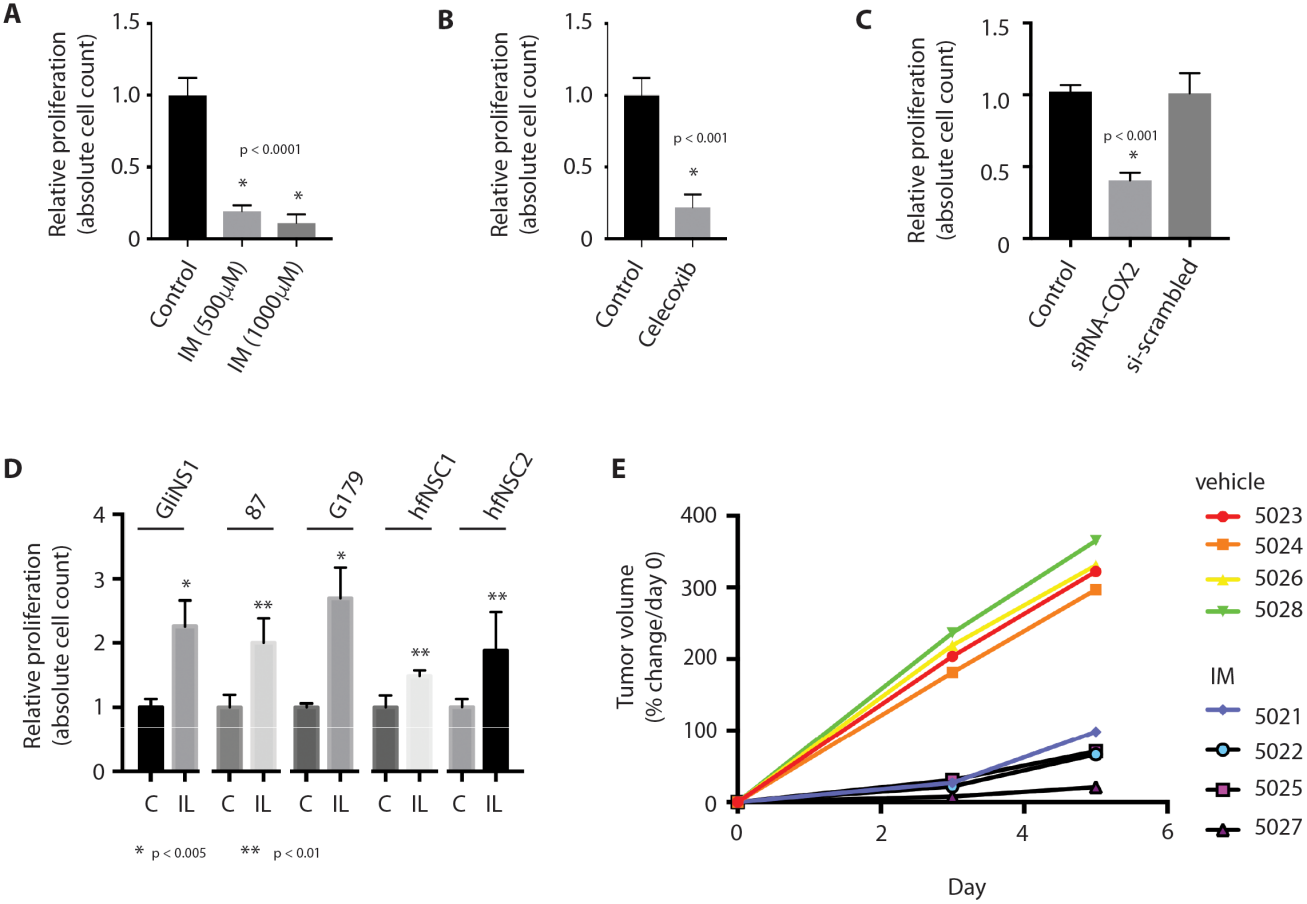
Inhibition of PGE2 production results in decreased GSC proliferation **(A)** Cox inhibition with indomethacin (IM) results in decreased proliferation in GSCs, as determined by absolute cell count. **(B, C)** Selective Cox-2 inhbition with celecoxib (50 μM) or siRNA against COX-2 (siRNA-COX2) results in decreased proliferation in GSCs, as determined by absolute cell count. **(D)** Treatment of GSCs with the synthetic PGE2 analog, iloprost (IL, 100 μM), promotes cell proliferation in GSCs (GliNS1, 2012087, G179) and NSCs (hfNSC1, hfNSC2), as determined by absolute cell count. * p <0.005, **p < 0.01. **(E)** Intratumoral inoculation with indomethacin (5021, 5022, 5025, 5027) impairs tumor growth in a heterotopic GBM xenograft model, compared to inoculation with control (5023, 5024, 5026, 5028). Data are represented as mean ± SEM.

As has been found in glioma cell lines and a mouse glioblastoma model, we postulated that Cox-2 activation could have a pro-proliferative effect on human GSCs through production of PGE2. Inhibition of PGE2 production in GSCs using the nonselective cyclooxeganse inhibitor, indomethacin, the specific Cox-2 inhibitor, celecoxib, or siRNA against COX-2, resulted in a significant decrease in cell proliferation, compared to treatment with vehicle or control siRNA (Figure 3A-3C). In contrast, treatment with the synthetic PGE2 analog, iloprost, resulted in enhanced proliferation in both GSCs and NSCs (Figure 3D). Finally, inoculation of a heterotopic patient-derived glioblastoma xenograft (PDX) with indomethacin resulted in slowed tumor growth, compared to tumors injected with a vehicle control (Figure 3E).

### Cox-2 regulates stem-ness in GSCs

**Figure 4:**
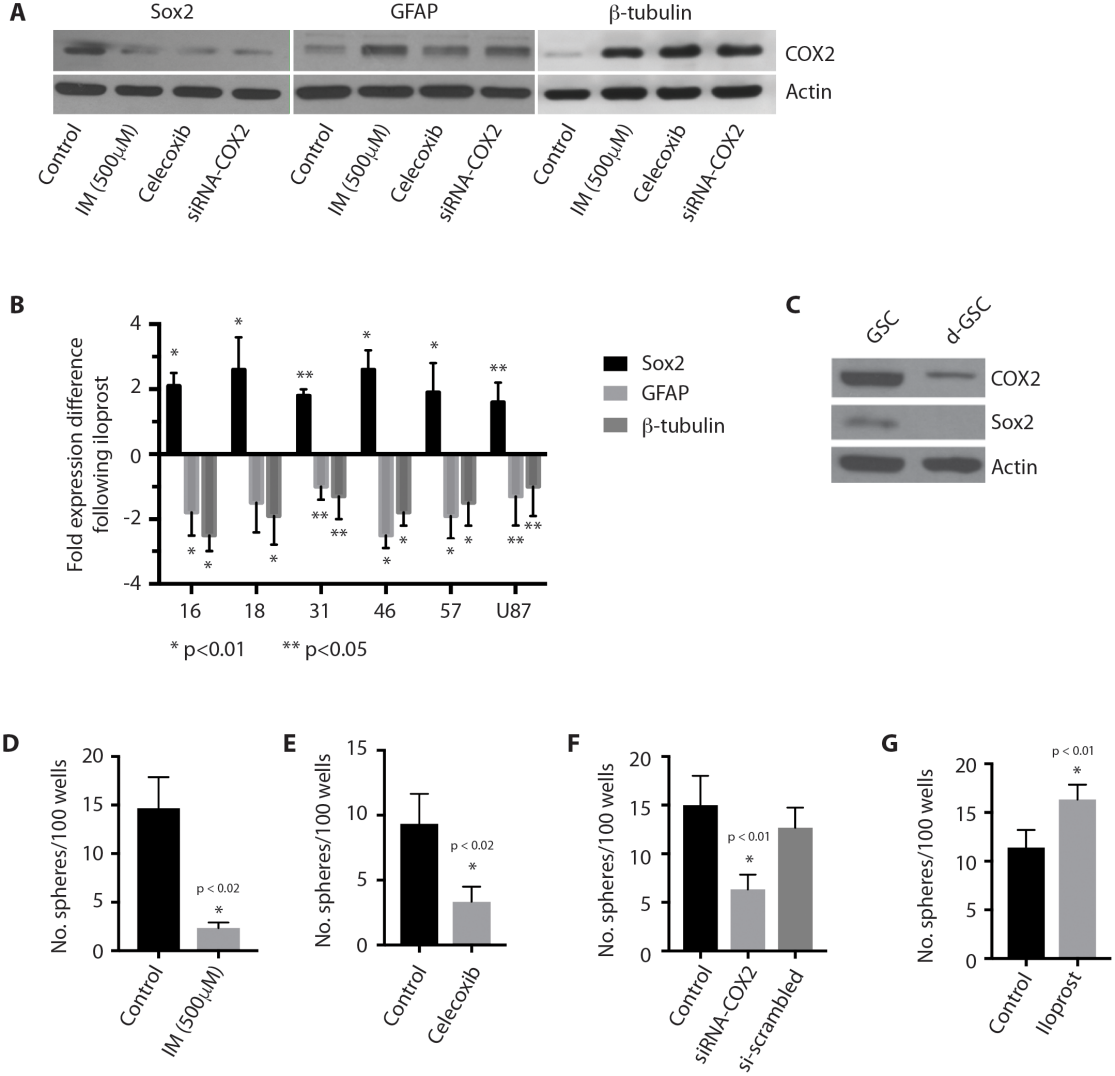
Cox-2 regulates stem-ness in GSCs **(A)** Coxinhibition with indomethacin or Cox-2 inhibition with celecoxib (50 μM) or siRNA-COX2 in GliNS1 GSCs results in decreased expression of the stem-ness marker, Sox2, and increased expression of the differentiation markers, GFAP and β-tubulin. **(B)** Treatment with iloprost (100 μM) results in increased expression of the stem-ness marker, Sox2, and decreased expression of the differentiation markers, GFAP and β-tubulin, in GSCs (2012016, 2012018, 201431, 2014046, 2014057), and the U87 glioblastoma cell line. * p <0.01, **p < 0.05. **(C)** Cox-2 expression in the GSC line GliNS1 following forced GSC differentiation by withdrawal of growth factors and treatment with serum. **(D-F)** Coxinhibition with indomethacin or Cox-2 inhibition with celecoxib (50 μM) or siRNA-COX2 results in decreased self-renewal in GliNS1 GSCs, as measured by the sphere formation assay. **(G)** Treatment of GliNS1 GSCs with iloprost (100 μM) results in increased GSC self-renewal, as measured by sphere formation. Data are represented as mean ± SEM.

We then sought to determine if Cox-2 signaling regulates the CSC identity in glioblastoma. Treatment of GSCs with indomethacin, celecoxib, or siRNA against Cox-2, resulted in a significant decrease in expression of the GSC marker Sox2, and an associated increase in expression of the differentiation markers, GFAP and β-tubulin (Figure 4A). Conversely, treatment of GSCs with iloprost resulted in a significant increase in expression of the GSC marker Sox2, and an associated decrease in expression of the differentiation markers, GFAP and β-tubulin, in multiple GSCs and the U87 glioblastoma cell line (Figure 4B). Cox-2 expression in glioma cells also decreased following forced differentiation (Figure 4C). Further, Cox-2 inhibition was accompanied by a decrease in GSC self-renewal, as approximated by sphere formation [26] (Figure 4D-4F), while treatment with the PGE2 analog, iloprost, promoted GSC sphere formation (Figure 4G). These findings suggest that Cox-2 signaling is necessary to maintain the cancer stem-like cell phenotype in glioblastoma.

### PGE2 activates the Wnt pathway in GSCs

**Figure 5:**
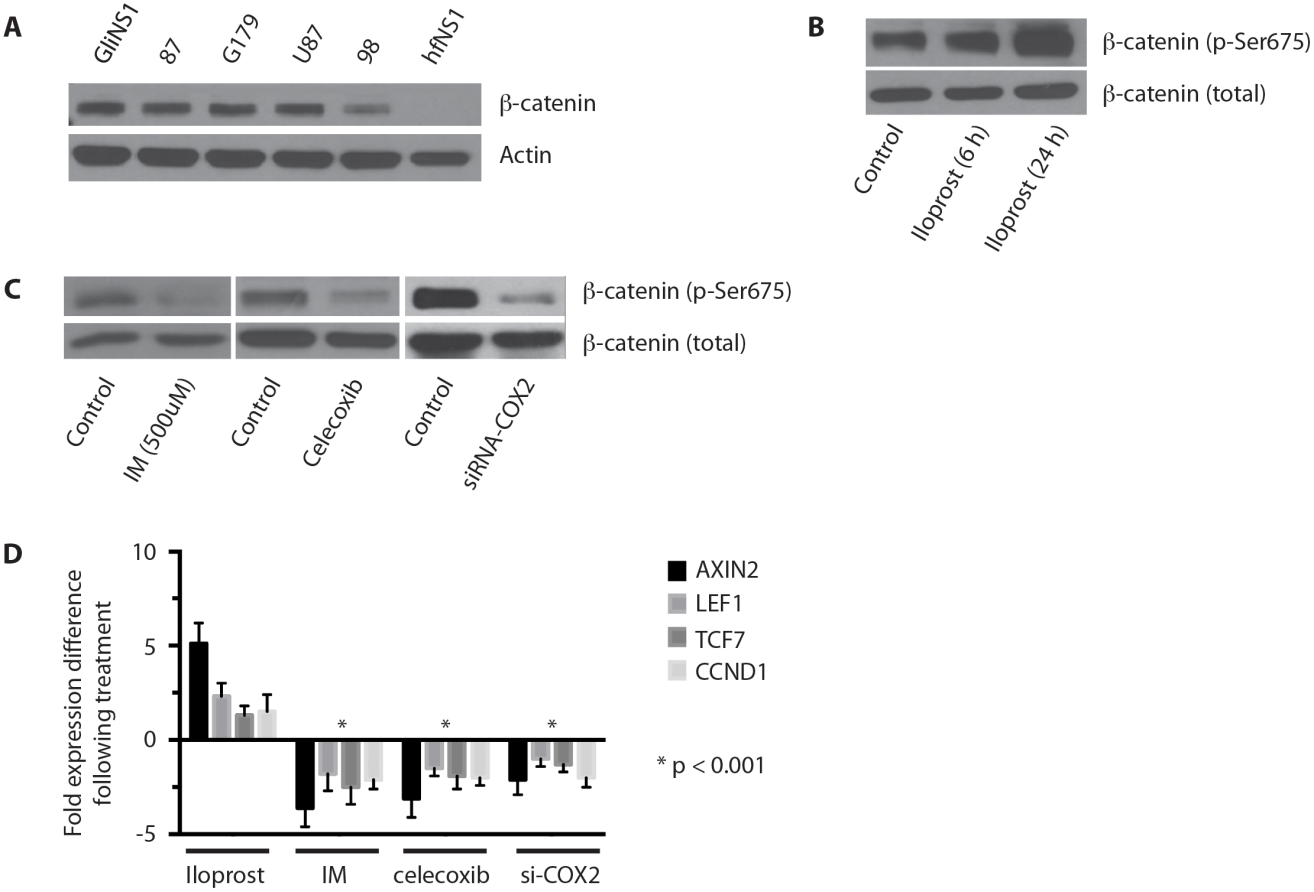
The Wnt pathway is activated in GSCs and enhanced by COX-2 signaling **(A)** Western blot analysis for β-catenin in untreated HGG-derived GSCs (GliNS1, 2012087, G179, 2015098), U87 MG, and human fetal neural stem cells (hfNS1). **(B)** Treatment of GliNS1 GSCs with iloprost (100 μM) results in increased expression of activated β-catenin (Ser675). **(C)** Coxinhibition with indomethacin or Cox-2 inhibition with celecoxib (50 μM) or siRNA-COX2 results in decreased expression of activated β-catenin in GliNS1 GSCs. **(D)** qRT-PCR for Wnt pathway targets AXIN2 and CCND1 and co-activators LEF1 and TCF7, following treatment of GliNS1 GSCs with the PGE2 agonist iloprost (100 μM) or Coxinhibition with indomethacin or Cox-2 inhibition with celecoxib (50 μM) or siRNA-COX2. * p < 0.001. Data are represented as mean ± SEM.

Investigation of the hematopoietic stem cell system has demonstrated a close association between the PGE2 and Wnt pathways in the regulation of HSC proliferation [20]. Wnt pathway activation has also been postulated to regulate symmetric cell division in normal NSCs [27, 28]. To determine if Wnt signaling regulates cell cycle entry in GSCs, we first looked for evidence of Wnt pathway activation in GSCs by immunoblotting for β-catenin accumulation in multiple GSC lines. We found β-catenin accumulation in multiple GSCs and glioma cell lines, but not in human fetal NSCs (Figure 5A).

We then sought to determine if PGE2 regulates the Wnt pathway in glioblastoma. Treatment of GSCs with iloprost resulted in an increase in β-catenin activation, as demonstrated by accumulation of phosphorylated β-catenin (Figure 5B) [29–31]. Conversely, PGE2 inhibition with indomethacin, celecoxib, or siRNA against COX-2, resulted in a significant decrease in phosphorylated β-catenin in GSCs (Figure 5C). To determine if PGE2 is relevant to canonical Wnt signaling, we then examined the effect of Cox-2 pathway modulation on the expression of LEF1 and TCF7, two Wnt pathway co-activators that mediate β-catenin binding to the genome [32], and the Wnt targets, AXIN2 and CCND1 [33, 34]. PGE2 induction with iloprost resulted in an increase in AXIN2, LEF1, TCF7, and CCND1 expression, while PGE2 inhibition with indomethacin, celecoxib, or siRNA against COX-2, resulted in a significant decrease in AXIN2, LEF1, TCF7, and CCND1 expression (Figure 5D). Our findings indicate the Cox-2 promotes activation of the Wnt signaling pathway in GSCs.

### Wnt pathway activation mediates self-renewal in GSCs

**Figure 6:**
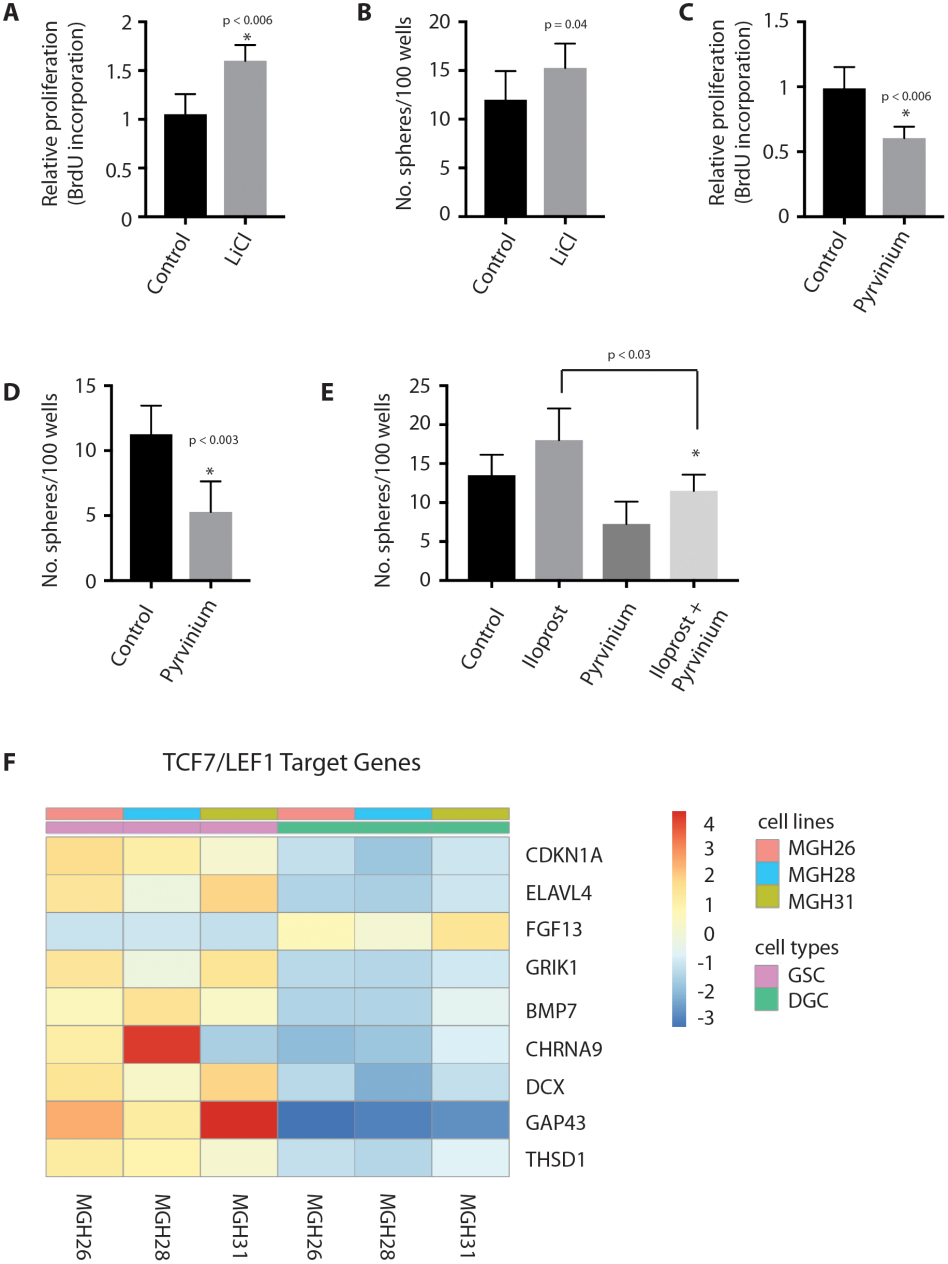
Wnt pathway activation mediates self-renewal in GSCs **(A, B)** Wnt activation in GSCs through treatment with the GSK3b-inhibitor lithium chloride (10 mM) promotes GliNS1 GSC proliferation and self-renewal, as determined by BrdU incorporation and sphere formation. **(C, D)** Wnt pathway inhibition with the casein kinase-1α potentiator pyrvinium (100 nM) results in decreased GliNS1 GSC proliferation and self-renewal, as determined by BrdU incorporation and sphere formation. **(E)** Wnt pathway inhibition with pyrvinium (10 nM) counteracts the stimulatory effect of PGE2 induction with iloprost (100 μM) on sphere formation in GliNS1 GSCs. **(F)** Wnt target genes are enriched in GSCs compared to differentiated glioma cells (DGCs) in the Bernstein dataset. Data are represented as mean ± SEM.

To determine the role of Wnt/β-catenin pathway in GSCs, we studied the effect of Wnt pathway activation and inhibition on GSCs *in vitro*. Wnt pathway activation with lithium chloride, which stabilizes endogenous β-catenin through inhibition of GSK3β [35], resulted in a significant increase in both GSC proliferation (Figure 6A) and self-renewal, as approximated by sphere formation (Figure 6B) *in vitro*. Conversely, treatment with the casein kinase-1α potentiator pyrvinium, which has been shown to inhibit the Wnt pathway inhibition by potentiating Axin-mediated degredation of β-catenin [36], resulted in a significant decrease in both GSC proliferation (Figure 6C) and sphere formation (Figure 6D). Wnt signaling had no apparent effect on GSC survival, as determined by expression of caspase-3 (data not shown). Interestingly, Wnt inhibition with pyrvinium abrogated the inductive effect of iloprost on GSC self-renewal, as approximated by sphere formation (Figure 6E), but not GSC proliferation (data not shown). As would be expected by these findings, analysis of GSCs and DGCs from the Bernstein dataset shows an enrichment of TCF7 and LEF1 target genes, as identified by Gene Ontology analysis, in GSCs compared to DGCs [37] (Figure 6F). Together, these data indicate that Wnt pathway activation promotes proliferation and self-renewal in GSCs, and that Wnt signaling may mediate the inductive effect of PGE2 on self-renewal [38].

### COX-2 inhibition enhances the effect of temozolomide in glioblastoma

**Figure 7:**
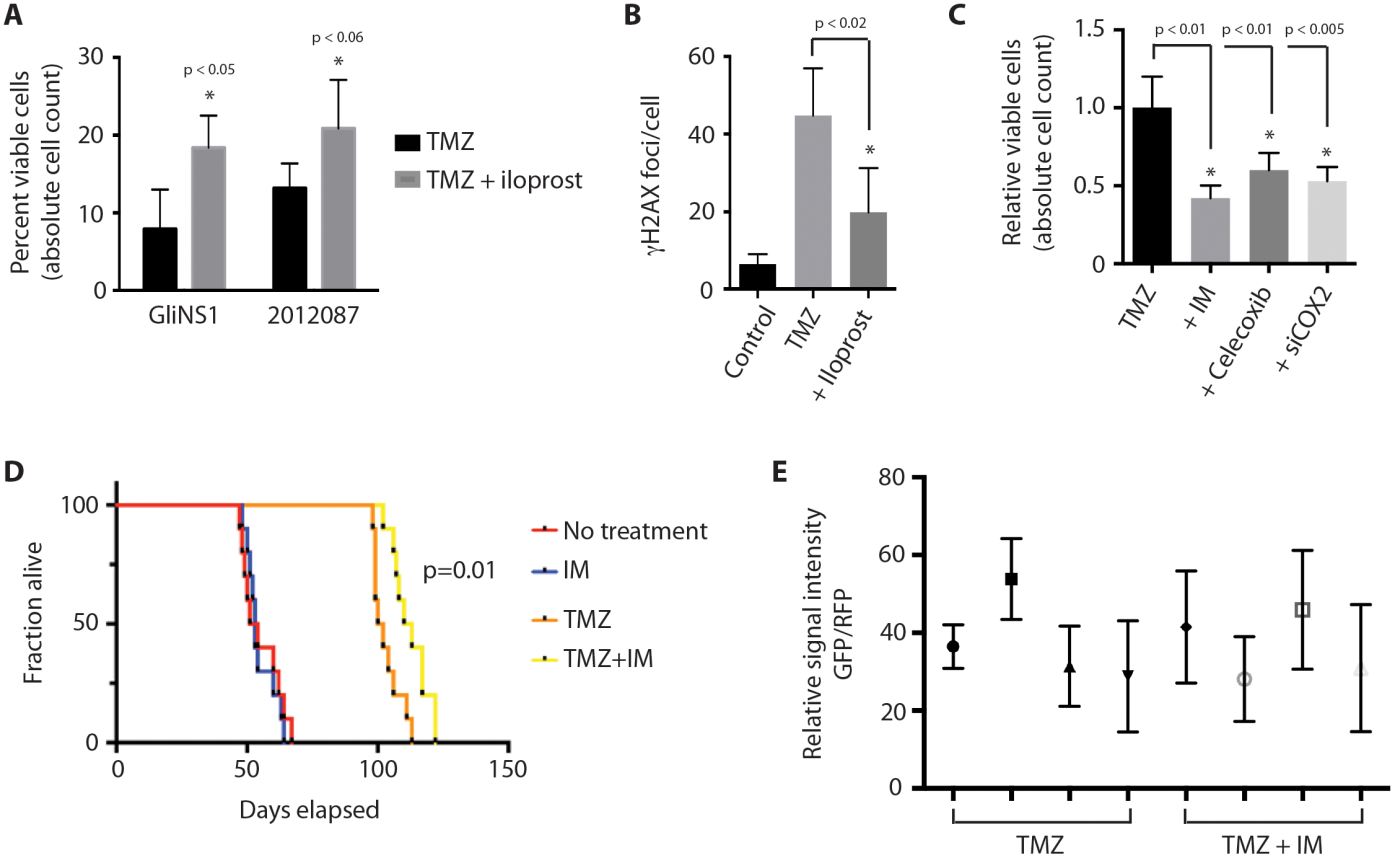
COX-2 inhibition enhances the cytotoxic effect of temozolomide in glioblastoma **(A)** Concurrent treatment of GSCs with iloprost (100 μM) protects GSCs (GliNS1, 2012087) from the cytotoxic effect of temozolomide (50 μM), as determined by absolute cell count. **(B)** γH2AX foci in GliNS1 GSCs treated with temozolomide (50 μM) with or without iloprost (100 μM). **(C)** Concurrent Cox inhibition with indomethacin (IM) or Cox-2 inhibition with celecoxib (50 μM) or siRNA against COX-2, potentiates the cytotoxic effect of temozolomide (50 μM) on GliNS1 GSCs. **(D)** Kaplan-Meier curve showing survival in a mouse glioblastoma orthotopic xeongraft. Treatment with the Cox inhibitor indomethacin had no direct effect on overall survival, but enhanced the survival benefit proffered by treatment with temozolomide. **(E)** No statistically significant difference (p=0.46) in the relative percentage of nuclear β-catenin-positive cells among surviving RFP-positive glioma cells in xenograft-harboring mice treated with temozolomide and indomethacin (n=4) compared to xenograft-harboring mice treated with temozolomide alone (n=4). Data are represented as mean ± SEM.

CSCs have been postulated to drive tumor recurrence following disease treatment through their preferential resistance to cytotoxic injury and cell death [3, 4, 39]. To determine if this hypothesis could be relevant to glioblastoma, we examined the effect of COX-2 signaling on cell survival following chemotoxic stress caused by exposure to the alkylating agent, temozolomide. Temozolomide is used as a first-line chemotherapeutic agent in patients with glioblastoma, and its use has been shown to increase overall and progression-free survival (Stupp et al). GSCs treated with iloprost and temozolomide demonstrated lower levels of cell death and DNA damage, as determined by absolute cell count and staining for phosphorylated histone H2AX (γH2AX), compared to GSCs treated with temozolomide alone (Figure 7A and 7B). Conversely, concurrent COX-2 inhibition with indomethacin, celecoxib, or siRNA against COX-2, increased the cytotoxic effect of temozolomide on GSCs, as demonstrated by absolute cell counts (Figure 7C). As would be predicted by these findings, indomethacin potentiated the effect of temozolomide chemotherapy on overall survival in a mouse orthotopic xenograft model (Figure 7D), though disappointingly the survival benefit was limited and non-sustained. Interestingly, we found evidence of sustained nuclear β-catenin staining within surviving glioma cells in xenograft-harboring mice treated with temozolomide and indomethacin compared to xenograft-harboring mice treated with temozolomide alone (Figure 7E), contrary to results that would be expected from our *in vitro* findings that COX inhibition results in decreased Wnt activation in glioma cells. The *in situ* findings support the presence of a COX-independent mechanism for activation of the Wnt pathway in glioma cells located within the brain microenvironment, and suggest a mechanism underlying the limited survival benefit conferred by concomitant COX inhibition therapy.

## DISCUSSION

In this study, we identify a cooperative role for Cox-2 and Wnt/β-catenin as mediators of the CSC phenotype in glioblastoma. Cox-2 is enriched in GSCs and promotes GSC proliferation and self-renewal through PGE2. Inhibition of Cox-2 directs glioma cells toward a differentiated phenotype and inhibits proliferation and self-renewal. As in the hematopoietic stem cell system, PGE2 activates the Wnt pathway in glioblastoma, resulting in increased levels of β-catenin and phosphorylated β-catenin. Finally, inhibition of the Wnt pathway abrogates the stimulatory effect of PGE2 on GSC self-renewal, suggesting that the modulatory effect of Cox-2 on GSC self-renewal is Wnt-dependent.

The Cox-2/PGE2 and Wnt/β-catenin pathways have been studied intensively as oncogenic drivers and are viewed as promising targets in several human cancers. Knockout of the COX-2 gene led to a marked reduction in the size and number of polyps in APCD716 mice, as did selective pharmacologic Cox-2 inhibition [40]. Exogenous Cox-2 expression sensitizes mouse skin to carcinogenesis [41], while COX-1 or -2 knockout inhibits skin papilloma formation [42]. Several epidemiologic studies have since shown an inverse correlation between the incidence of colon cancer and the use of nonsteroidal anti-inflammatory drugs (NSAIDs), which inhibit prostaglandin synthesis [43–45]. Similarly, WNT1 overexpression induced by a proviral insertion at the *Wnt1* locus induces spontaneous mammary hyperplasia and tumours in mice [46, 47], and *Wnt1* transgenic mice similarly develop mammary tumours, suggesting a causative role for WNT1 in breast cancer tumorigenesis [48]. Activating Wnt mutations have also been found in colorectal cancer, Wilms tumors, hepatocellular carcinoma (HCC) [49], medulloblastoma [50] and ovarian cancer [51].

Zon and colleagues have previously identified an interaction between the Wnt and prostaglandin signaling networks in murine stem and progenitor populations in the hematopoietic system [20]. In the hematopoietic system, PGE2 modified the Wnt signaling cascade at the level of β-catenin degradation through a cAMP/PKA-mediated phosphorylation event that stabilizes β-catenin. Similarly, Ferrari and colleagues has found that Wnt/β-catenin signaling enhances the transcription of COX2 in gastric cancer cells by binding to a TCF/LEF-response element in the COX2 promoter [52].

Our results demonstrate a role for Cox-2/PGE2 signaling as a regulator of the GSC identity, and reinforce a therapeutic rationale for but possible limitation of Cox-2 inhibitors in glioblastoma. Chemical or genetic inhibition of PGE2 production results in differentiation of GSCs and inhibition of GSC proliferation and self-renewal. PGE2 production also results in activation of the Wnt/β-catenin pathway, which in turn stimulates GSC proliferation and self-renewal. Interestingly, Wnt inhibition abrogates the positive effect of PGE2 induction on GSC sphere formation, suggesting the stimulatory effect of COX-2 on GSC self-renewal is mediated through the Wnt pathway. This finding is intriguing, as Wnt signaling has also been found to direct symmetric cell division in adult mouse NSCs [21], and has been found to be a critical target of PGE2 during HSC specification and regeneration [20]. Our findings also suggest that cell-extrinsic, PGE2-independent activation of the Wnt pathway could mitigate a therapeutic benefit of Cox-2 inhibition in glioblastoma. It is tempting to speculate that the lack of significant clinical efficacy of COX-2 inhibition in our animal data and in the clinical experience with patients with glioblastoma may be explained by our finding *in situ* of increased Wnt activation in the setting of IM therapy. It may very well be that concurrent use of Wnt inhibitors will ameliorate this deficit.

The Wnt signaling pathway has recently been described to be constitutively activated in a subgroup of glioblastomas, and it is possible that the kinetics of mitosis in these tumors favors self-renewal rather than proliferation as the vector for tumor expansion [53, 54]. It is intriguing to speculate that Wnt signaling could play a role in GBM treatment resistance and tumor recurrence [55, 56], and in this context, underlie resistance to Cox inhibition therapy. Our data also reinforce prior findings that proliferation and self-renewal are molecularly distinct processes in glioblastoma, and prompt further study to understand the role of self-renewal in glioblastoma development, progression and recurrence.

### Experimental procedures

The experimental work included in this manuscript was performed at the University of Toronto, Toronto, Ontario, and Washington University, St. Louis, Missouri. All experiments were performed in accordance with the relevant guidelines and regulations of these two institutions.

### Immunohistochemistry in human glioblastoma specimens

Human glioblastoma specimens and clinical data were obtained from the St. Michael Hospital Brain Tumour Biobank and Brain Tumour Research Centre, Hospital for Sick Children, following approval by the Institutional Research Ethics Boards of both institutions. All samples were de-identified before analysis. Tissue samples were preserved in 10% formalin, dehydrated and embedded in paraffin. Five μm sections were immunostained for COX-2 (Abcam 1:1000), with antigen retrieval using pressure cooking in 10uM citrate buffer at pH6.0, secondary HRP anti-Rabbit IgG (Vector lab 1:250), and detection using DAB Substrate kit (Vector lab). Nuclei were counterstained with DAPI (0.002 mg/ml Sigma) for 10 minutes, washed and mounted with Vectashield mounting medium (Vector Laboratories, Inc.). Images were acquired with a Quorum Spinning Disk Confocal Microscope (Olympus) running Volocity software (Perkin Elmer).

### Analysis of COX2 expression in glioblastoma from TCGA

TCGA GBM RNAseq data was accessed using *TCGA-Assembler* [57]. RSEM normalized reads for COX2 were extracted and plotted using custom scripts in *R*. Differential expression between molecular subgroups were assessed using a Welch’s two-sample t-test.

### Cell culture

The generation of adherent human GSC cultures has been described previously [25]. Informed consent was obtained from patients for use of human tissue and cells, and all human tissue-related protocols used in this study were approved by the Review Ethics Board (University of Toronto). In brief, tumor samples obtained directly from surgery were dissociated by mincing and incubation in Accutase (Sigma-Aldrich) for 20–60 min at 37C. Cell suspensions were passed through a 70-mm cell strainer (Falcon) and plated using Ndiff RHB-A media (Stem Cell Sciences) with EGF and FGF-2 (PeproTech) (hereafter called GSC media) each at 20 ng/ml, on polyornithine- and laminin- (Sigma- Aldrich) coated Primaria dishes/flasks (BD Biosciences). Media were replaced with half fresh GSC media every 2–3 days. Cells were routinely used between passages 5 and 20. U87 cells were cultured in DMEM with 10% fetal bovine serum (FBS) and penicillin/streptomycin (Life Technologies). All cell lines were incubated at 37°C with 5% CO2.

### Neurosphere and proliferation assays

GSCs were diluted to a concentration of 1 cell/100 μL and distributed to a 96-well plate. 3 plates (288 wells) were used per experiment. Phase microscopy was used to confirm that each well contained a single cell. Then, 7–10 days later, the number of wells containing spheres was manually counted and used to calculate the frequency of self-renewing GSCs. For proliferation studies, cells were incubated with BrdU and assayed according to protocol (BrdU Cell Proliferation Kit, Cell Signaling), or assessed by manual counting.

### Xenotransplantation

Animal studies were performed in accordance with the Toronto Centre for Phenogenomics (TCP) institutional animal protocols and approved by the Animal Studies Committee of the University of Toronto. For heterotopic (flank) model studies, 50,000 cells were injected in the mouse flank of approximately 6-week-old NOD-SCID mice. Drug administration in the flank xenograft model was performed through direct intratumoral injection using a volume of 200 uL per injection of either normal saline (0.9%) or 1.5 mM indomethacin. A total of five intratumoral injections were given every other day. The mice were checked daily and sacrificed 24 hours after the last injection. For orthotopic (intracranial) model studies, 25,000 cells were injected stereotactically into the right putamen of approximately 6-week-old NOD-SCID mice. The coordinates used were: 1 mm rostral to bregma, 2 mm lateral, and 2.5 mm deep.

### Western blotting and protein quantification

Cells were washed with cold PBS and lysed. Cell lysates were shaken at 4°C for 10 min and centrifuged at 14,000 × g for 10 min. Protein concentrations in the supernatants were quantified using the BCA protein assay. Fifteen micrograms of protein were separated on 10% acrylamide/bisacrylamide gels and transferred to PVDF membranes. PVDF membranes and blocked with 5% (w/v) skim milk in PBS/0.1% Tween-20 for 1 h at room temperature. Membranes were shaken with primary antibodies at 4°C overnight, then washed and incubated with secondary antibodies (Cell Signaling) for 1 hr at room temperature. ECL (Bio-Rad) was used for detection of proteins from Western blots. The following primary antibodies were used: β-actin (Cell Signaling, 1:10,000), Cox-2 (Abcam, 1:1000), β-catenin (Cell Signaling Technology, 1:1000), phospho-β-catenin Ser675 (Cell Signaling Technology, 1:1000), and cleaved caspase 3 (Cell Signaling Technology, 1:1000). Protein quantification was performed by densitometry (Image Studio, LI-COR). Protein levels were normalized for β-actin.

### ELISA assay

ELISA assays were performed on conditioned medium using a PGE_2_ EIA kit (R&D) according to the manufacturer’s instructions.

### Quantitative real-time PCR

Total RNA was extracted from cells in culture using an RNAEasy Kit according to the manufacturer’s instructions (Qiagen), and cDNA synthesis was performed using SuperScript II (Invitrogen). Assays were performed using SYBR Green PCR Master Mix (Applied Biosystems) with a StepOne Real Time PCR System. Predesigned primers were purchased from IDT. Gene transcript levels were calculated using the ΔΔCt method.

### Short interfering RNA knockdown

Predesigned short interfering (si)RNAs targeting human COX-2 and scrambled control siRNAs were purchased from Qiagen. Two separate siRNAs targeting different sequences within COX-2 were used to transfect U87 cells using Lipofectamine 2000 (Invitrogen) versus scrambled control siRNA. Cells were harvested 48 hours post-transfection for analysis of protein and RNA levels.

### TCGA data analysis

TCGA level 3 data was gathered using *TCGA-Assembler* retrieving RNA-seqV2 data and clinical data for glioblastoma and low-grade glioma as above. RSEM normalized expression values were used to group glioblastomas by COX2 expression. Glioblastomas expressing COX2 in the first quartile were assigned as COX2-high expressing and GBMs with COX2 expression in the 4^th^ quartile was assigned COX2-low expressing. Boxplots of TCF/LEF target genes were performed in R using RSEM normalized expression. Statistical significance was determined with student’s t-test using a p-value cut-off of 0.05.

### RNA-seq data analysis

RNA-seq fastq data was obtained via accession no. GSE57872. Paired-end 25 bp reads were mapped to UCSC human genome (hg38) by STAR (version 2.5 using default parameters). Differential expression of genes was performed using DESeq2 [58]. Log2 transformed count matrix for TCF/LEF target genes with adjusted p-value < 0.05 were plotted in a heatmap with R package “*pheatmap”*.

### Xenograft tissue preparation and immunohistochemistry

Adult mice (Jackson labs) were anesthetised with Avertin (2, 2, 2-tribromoethanol, Sigma) and perfused with 4% paraformaldehyde. Brains were post-fixed overnight and cryoprotected in 20% sucrose at 4°C until sectioning. Coronal, cryostat sections (20 μm) were mounted on Superfrost Plus slides (Fisher Scientific) and immunostained. Briefly, brain sections were rehydrated for 5 min and permeabilized using 0.3% Triton in PBS for 20 min, rinsed with PBS, and blocked with 1% BSA containing 0.3% Triton in PBS at room temperature. Sections were incubated with rabbit β-catenin (abcam, 1:100) primary antibody overnight at 4°C. The following day, slides were washed with PBS and incubated with Alexa Fluor 488 goat-anti-rabbit (1:400, Invitrogen) secondary antibody for 1 hr at room temperature. Nuclei were counterstained with TP-PRO-3 Iodide (ThermoFisher Scientific), washed and mounted with Vecta Mount mounting medium (Vector Laboratories, Inc.). Images were acquired with a Quorum Spinning Disk Confocal Microscope (Olympus) running Volocity software (Perkin Elmer). Quantification of GFP signal within RFP-positive cells was performed using ImageJ (https://imagej.nih.gov/ij/).

### Statistics

All images are representative of results from three independent experiments unless otherwise stated. Statistical analyses were performed with Prism 7.0b (GraphPad), Excel (Microsoft), or R Version 3.1.1 software. The unpaired Student’s t-test was used for comparisons in experiments with only two groups. In experiments with more than two comparison groups, ANOVA was performed. Record of statistical significance has been included in the figure or figure legends.

## SUPPLEMENTARY MATERIALS FIGURE



## References

[1] Stupp R, Mason WP, van den Bent MJ, Weller M, Fisher B, Taphoorn MJ, Belanger K, Brandes AA, Marosi C, Bogdahn U, Curschmann J, Janzer RC, Ludwin SK, Gorlia T, Allgeier A, Lacombe D (2005). Radiotherapy plus concomitant and adjuvant temozolomide for glioblastoma. N Engl J Med.

[2] Singh SK, Hawkins C, Clarke ID, Squire JA, Bayani J, Hide T, Henkelman RM, Cusimano MD, Dirks PB (2004). Identification of human brain tumour initiating cells. Nature.

[3] Bao S, Wu Q, McLendon RE, Hao Y, Shi Q, Hjelmeland AB, Dewhirst MW, Bigner DD, Rich JN (2006). Glioma stem cells promote radioresistance by preferential activation of the DNA damage response. Nature.

[4] Chen J, Li Y, Yu TS, McKay RM, Burns DK, Kernie SG, Parada LF (2012). A restricted cell population propagates glioblastoma growth after chemotherapy. Nature.

[5] Cheng WY, Kandel JJ, Yamashiro DJ, Canoll P, Anastassiou D (2012). A multi-cancer mesenchymal transition gene expression signature is associated with prolonged time to recurrence in glioblastoma. PLoS One.

[6] Wang J, Wakeman TP, Lathia JD, Hjelmeland AB, Wang XF, White RR, Rich JN, Sullenger BA (2010). Notch promotes radioresistance of glioma stem cells. Stem Cells.

[7] Clement V, Sanchez P, de Tribolet N, Radovanovic I (2007). Ruiz i Altaba A. HEDGEHOG-GLI1 signaling regulates human glioma growth, cancer stem cell self-renewal, and tumorigenicity. Currt Biol.

[8] Jin X, Jeon HM, Jin X, Kim EJ, Yin J, Jeon HY, Sohn YW, Oh SY, Kim JK, Kim SH, Jung JE, Kwak S, Tang KF (2016). The ID1-CULLIN3 axis regulates intracellular SHH and WNT signaling in glioblastoma stem cells. Cell Rep.

[9] Dolma S, Selvadurai HJ, Lan X, Lee L, Kushida M, Voisin V, Whetstone H, So M, Aviv T, Park N, Zhu X, Xu C, Head R (2016). Inhibition of dopamine receptor D4 impedes autophagic flux, proliferation, and survival of glioblastoma stem cells. Cancer Cell.

[10] Dandekar DS, Lokeshwar BL (2004). Inhibition of cyclooxygenase (COX)-2 expression by Tet-inducible COX-2 antisense cDNA in hormone-refractory prostate cancer significantly slows tumor growth and improves efficacy of chemotherapeutic drugs. Clinical Cancer Res.

[11] Kishi K, Petersen S, Petersen C, Hunter N, Mason K, Masferrer JL, Tofilon PJ, Milas L (2000). Preferential enhancement of tumor radioresponse by a cyclooxygenase-2 inhibitor. Cancer Res.

[12] Tsujii M, Kawano S, DuBois RN (1997). Cyclooxygenase-2 expression in human colon cancer cells increases metastatic potential. Proc Natl Acad Sci U S A.

[13] Tsujii M, Kawano S, Tsuji S, Sawaoka H, Hori M, DuBois RN (1998). Cyclooxygenase regulates angiogenesis induced by colon cancer cells. Cell.

[14] Shono T, Tofilon PJ, Bruner JM, Owolabi O, Lang FF (2001). Cyclooxygenase-2 expression in human gliomas: prognostic significance and molecular correlations. Cancer Res.

[15] Sareddy GR, Geeviman K, Ramulu C, Babu PP (2012). The nonsteroidal anti-inflammatory drug celecoxib suppresses the growth and induces apoptosis of human glioblastoma cells via the NF-kappaB pathway. J Neurooncol.

[16] Sharma V, Dixit D, Ghosh S, Sen E (2011). COX-2 regulates the proliferation of glioma stem like cells. Neurochem Int.

[17] Cook PJ, Thomas R, Kingsley PJ, Shimizu F, Montrose DC, Marnett LJ, Tabar VS, Dannenberg AJ, Benezra R (2016). Cox-2-derived PGE2 induces Id1-dependent radiation resistance and self-renewal in experimental glioblastoma. Neuro Oncol.

[18] Xu K, Wang L, Shu HK (2014). COX-2 overexpression increases malignant potential of human glioma cells through Id1. Oncotarget.

[19] North TE, Goessling W, Walkley CR, Lengerke C, Kopani KR, Lord AM, Weber GJ, Bowman TV, Jang IH, Grosser T, Fitzgerald GA, Daley GQ, Orkin SH, Zon LI (2007). Prostaglandin E2 regulates vertebrate haematopoietic stem cell homeostasis. Nature.

[20] Goessling W, North TE, Loewer S, Lord AM, Lee S, Stoick-Cooper CL, Weidinger G, Puder M, Daley GQ, Moon RT, Zon LI (2009). Genetic interaction of PGE2 and Wnt signaling regulates developmental specification of stem cells and regeneration. Cell.

[21] Piccin D, Morshead CM (2011). Wnt signaling regulates symmetry of division of neural stem cells in the adult brain and in response to injury. Stem Cells.

[22] Ying QL, Wray J, Nichols J, Batlle-Morera L, Doble B, Woodgett J, Cohen P, Smith A (2008). The ground state of embryonic stem cell self-renewal. Nature.

[23] Moon Y, Bottone FG, McEntee MF, Eling TE (2005). Suppression of tumor cell invasion by cyclooxygenase inhibitors is mediated by thrombospondin-1 via the early growth response gene Egr-1. Mol Cancer Therapeut.

[24] Pattabiraman DR, Weinberg RA (2014). Tackling the cancer stem cells - what challenges do they pose?. Nat Rev Drug Discov.

[25] Pollard SM, Yoshikawa K, Clarke ID, Danovi D, Stricker S, Russell R, Bayani J, Head R, Lee M, Bernstein M, Squire JA, Smith A, Dirks P (2009). Glioma stem cell lines expanded in adherent culture have tumor-specific phenotypes and are suitable for chemical and genetic screens. Stem Cell.

[26] Pastrana E, Silva-Vargas V, Doetsch F (2011). Eyes wide open: a critical review of sphere-formation as an assay for stem cells. Cell Stem Cell.

[27] Kalani MY, Cheshier SH, Cord BJ, Bababeygy SR, Vogel H, Weissman IL, Palmer TD, Nusse R (2008). Wnt-mediated self-renewal of neural stem/progenitor cells. Proc Natl Acad of Sci U S A.

[28] Piccin D, Morshead CM (2011). Wnt signaling regulates symmetry of division of neural stem cells in the adult brain and in response to injury. Stem Cells.

[29] Luckert K, Gujral TS, Chan M, Sevecka M, Joos TO, Sorger PK, Macbeath G, Potz O (2012). A dual array-based approach to assess the abundance and posttranslational modification state of signaling proteins. Sci Signal.

[30] Selamat W, Tay PL, Baskaran Y, Manser E (2015). The Cdc42 effector kinase PAK4 localizes to cell-cell junctions and contributes to establishing cell polarity. PLoS One.

[31] Spirli C, Locatelli L, Morell CM, Fiorotto R, Morton SD, Cadamuro M, Fabris L, Strazzabosco M (2013). Protein kinase A-dependent pSer(675) -beta-catenin, a novel signaling defect in a mouse model of congenital hepatic fibrosis. Hepatology.

[32] Cadigan KM, Waterman ML (2012). TCF/LEFs and Wnt signaling in the nucleus. Cold Spring Harb Perspect Biol.

[33] Jho EH, Zhang T, Domon C, Joo CK, Freund JN, Costantini F (2002). Wnt/beta-catenin/Tcf signaling induces the transcription of Axin2, a negative regulator of the signaling pathway. Mol Cell Biol.

[34] Ford CE, Jary E, Ma SS, Nixdorf S, Heinzelmann-Schwarz VA, Ward RL (2013). The Wnt gatekeeper SFRP4 modulates EMT, cell migration and downstream Wnt signalling in serous ovarian cancer cells. PLoS One.

[35] Clement-Lacroix P, Ai M, Morvan F, Roman-Roman S, Vayssiere B, Belleville C, Estrera K, Warman ML, Baron R, Rawadi G (2005). Lrp5-independent activation of Wnt signaling by lithium chloride increases bone formation and bone mass in mice. Proc Natl Acad Sci U S A.

[36] Thorne CA, Hanson AJ, Schneider J, Tahinci E, Orton D, Cselenyi CS, Jernigan KK, Meyers KC, Hang BI, Waterson AG, Kim K, Melancon B, Ghidu VP (2010). Small-molecule inhibition of Wnt signaling through activation of casein kinase 1alpha. Nat Chem Biol.

[37] Suva ML, Rheinbay E, Gillespie SM, Patel AP, Wakimoto H, Rabkin SD, Riggi N, Chi AS, Cahill DP, Nahed BV, Curry WT, Martuza RL, Rivera MN (2014). Reconstructing and reprogramming the tumor-propagating potential of glioblastoma stem-like cells. Cell.

[38] Deleyrolle LP, Ericksson G, Morrison BJ, Lopez JA, Burrage K, Burrage P, Vescovi A, Rietze RL, Reynolds BA (2011). Determination of somatic and cancer stem cell self-renewing symmetric division rate using sphere assays. PLoS One.

[39] O'Brien CA, Kreso A, Ryan P, Hermans KG, Gibson L, Wang Y, Tsatsanis A, Gallinger S, Dick JE (2012). ID1 and ID3 regulate the self-renewal capacity of human colon cancer-initiating cells through p21. Cancer Cell.

[40] Oshima M, Dinchuk JE, Kargman SL, Oshima H, Hancock B, Kwong E, Trzaskos JM, Evans JF, Taketo MM (1996). Suppression of intestinal polyposis in Apc delta716 knockout mice by inhibition of cyclooxygenase 2 (COX-2). Cell.

[41] Muller-Decker K, Neufang G, Berger I, Neumann M, Marks F, Furstenberger G (2002). Transgenic cyclooxygenase-2 overexpression sensitizes mouse skin for carcinogenesis. Proc Natl Acad Sci U S A.

[42] Tiano HF, Loftin CD, Akunda J, Lee CA, Spalding J, Sessoms A, Dunson DB, Rogan EG, Morham SG, Smart RC, Langenbach R (2002). Deficiency of either cyclooxygenase (COX)-1 or COX-2 alters epidermal differentiation and reduces mouse skin tumorigenesis. Cancer Res.

[43] Friis S, Riis AH, Erichsen R, Baron JA, Sorensen HT (2015). Low-dose aspirin or nonsteroidal anti-inflammatory drug use and colorectal cancer risk: a population-based, case-control Study. Ann Intern Med.

[44] Ng K, Meyerhardt JA, Chan AT, Sato K, Chan JA, Niedzwiecki D, Saltz LB, Mayer RJ, Benson AB, Schaefer PL, Whittom R, Hantel A, Goldberg RM (2015). Aspirin and COX-2 inhibitor use in patients with stage III colon cancer. J Natl Cancer Inst.

[45] Johnson CC, Jankowski M, Rolnick S, Yood MU, Alford SH (2017). Influence of NSAID Use Among Colorectal Cancer Survivors on Cancer Outcomes. Am J Clin Oncol.

[46] Nusse R, van Ooyen A, Cox D, Fung YK, Varmus H (1984). Mode of proviral activation of a putative mammary oncogene (int-1) on mouse chromosome 15. Nature.

[47] Nusse R, Varmus HE (1982). Many tumors induced by the mouse mammary tumor virus contain a provirus integrated in the same region of the host genome. Cell.

[48] Tsukamoto AS, Grosschedl R, Guzman RC, Parslow T, Varmus HE (1988). Expression of the int-1 gene in transgenic mice is associated with mammary gland hyperplasia and adenocarcinomas in male and female mice. Cell.

[49] Breuhahn K, Longerich T, Schirmacher P (2006). Dysregulation of growth factor signaling in human hepatocellular carcinoma. Oncogene.

[50] Zurawel RH, Chiappa SA, Allen C, Raffel C (1998). Sporadic medulloblastomas contain oncogenic beta-catenin mutations. Cancer Res.

[51] Palacios J, Gamallo C (1998). Mutations in the beta-catenin gene (CTNNB1) in endometrioid ovarian carcinomas. Cancer Res.

[52] Nunez F, Bravo S, Cruzat F, Montecino M, De Ferrari GV (2011). Wnt/beta-catenin signaling enhances cyclooxygenase-2 (COX2) transcriptional activity in gastric cancer cells. PLoS One.

[53] Kim KH, Seol HJ, Kim EH, Rheey J, Jin HJ, Lee Y, Joo KM, Lee J, Nam DH (2013). Wnt/beta-catenin signaling is a key downstream mediator of MET signaling in glioblastoma stem cells. Neuro Oncol.

[54] Morris LG, Kaufman AM, Gong Y, Ramaswami D, Walsh LA, Turcan S, Eng S, Kannan K, Zou Y, Peng L, Banuchi VE, Paty P, Zeng Z (2013). Recurrent somatic mutation of FAT1 in multiple human cancers leads to aberrant Wnt activation. Nat Genet.

[55] Tomasetti C, Levy D (2010). Role of symmetric and asymmetric division of stem cells in developing drug resistance. Proc Natl Acad Sci U S A.

[56] Li X, Lewis MT, Huang J, Gutierrez C, Osborne CK, Wu MF, Hilsenbeck SG, Pavlick A, Zhang X, Chamness GC, Wong H, Rosen J, Chang JC (2008). Intrinsic resistance of tumorigenic breast cancer cells to chemotherapy. J Natl Cancer Inst.

[57] Zhu Y, Qiu P, Ji Y (2014). TCGA-assembler: open-source software for retrieving and processing TCGA data. Nat Methods.

[58] Love MI, Huber W, Anders S (2014). Moderated estimation of fold change and dispersion for RNA-seq data with DESeq2. Genome Biol.

