# Supplementary material for: Aberrantly activated Cox-2 and Wnt signaling interact to maintain cancer stem cells in glioblastoma
Source: Oncotarget. 2017 Jul 17;8(47):82217–30. doi: 10.18632/oncotarget.19283 (PMC5669884; doi:10.18632/oncotarget.19283)
Supplement: Supplementary file 1 [file oncotarget-08-82217-s001.pdf]

Aberrantly activated Cox-2 and Wnt signaling interact to maintain cancer stem cells in glioblastoma

SUPPLEMENTARY MATERIALS

Tissue preparation and immunohistochemistry

Adult CD1 mice (Jackson labs) were anesthetised with Avertin (2, 2, 2-tribromoethanol, Sigma) and perfused with 4% paraformaldehyde. Brains were post-fixed overnight and cryoprotected in 20% sucrose at 4°C until sectioning. Coronal, cryostat sections (20 μm) were mounted on Superfrost Plus slides (Fisher Scientific) and immunostained. Briefly, brain sections were rehydrated for 5 min and permeabilized using 0.3% Triton in PBS for 20 min, rinsed with PBS, and blocked with 1% BSA containing 0.3% Triton in PBS at room temperature. Sections were incubated with rabbit anti-COX-2 (1:200, AB15191, Abcam) primary antibody overnight at 4°C. The following day, slides were washed with PBS and incubated with Alexa Fluor 488 goat-anti-rabbit (1:400, Invitrogen) secondary antibody for 1 hr at room temperature. Nuclei were counterstained with Hoechst 33258 (0.000725 mg/ml Sigma) for 10 minutes, washed and mounted with Vecta Mount mounting medium (Vector Laboratories, Inc.). Sections were visualized and imaged using Zen 2011 software and a Zeiss two-photon microscope.

Surgical preparations and stroke injury model for COX-2 positive control

Adult male C57BL/6J of 6-8 weeks of age were anesthetized with isoflluorane (5% induction and 2% maintenance) followed by a subcutaneous administration of ketoprofen (a volume of 0.1 ml per 10g body weight and 0.5 mg/ml in dosage). Alcohol and betadine was applied to the skin before making an incision. A total of four holes were drilled into the skull. To induce ischemic lesions in the medial prefrontal cortex, endothelin(ET)-1 (800 picomolar) was injected bilaterally at the following cortical coordinates: (1) anterior-posterior (AP)= +2.2 mm from Bregma; mediolateral (ML)= ±0.4 mm from midline; dorsoventral (DV)=2.4 mm, and (2) AP= +1.5 mm from Bregma, ML= 0.4 mm from midline, DV=2.6 mm below the skull surface. The needle was carefully withdrawn 3 minutes after each injection.

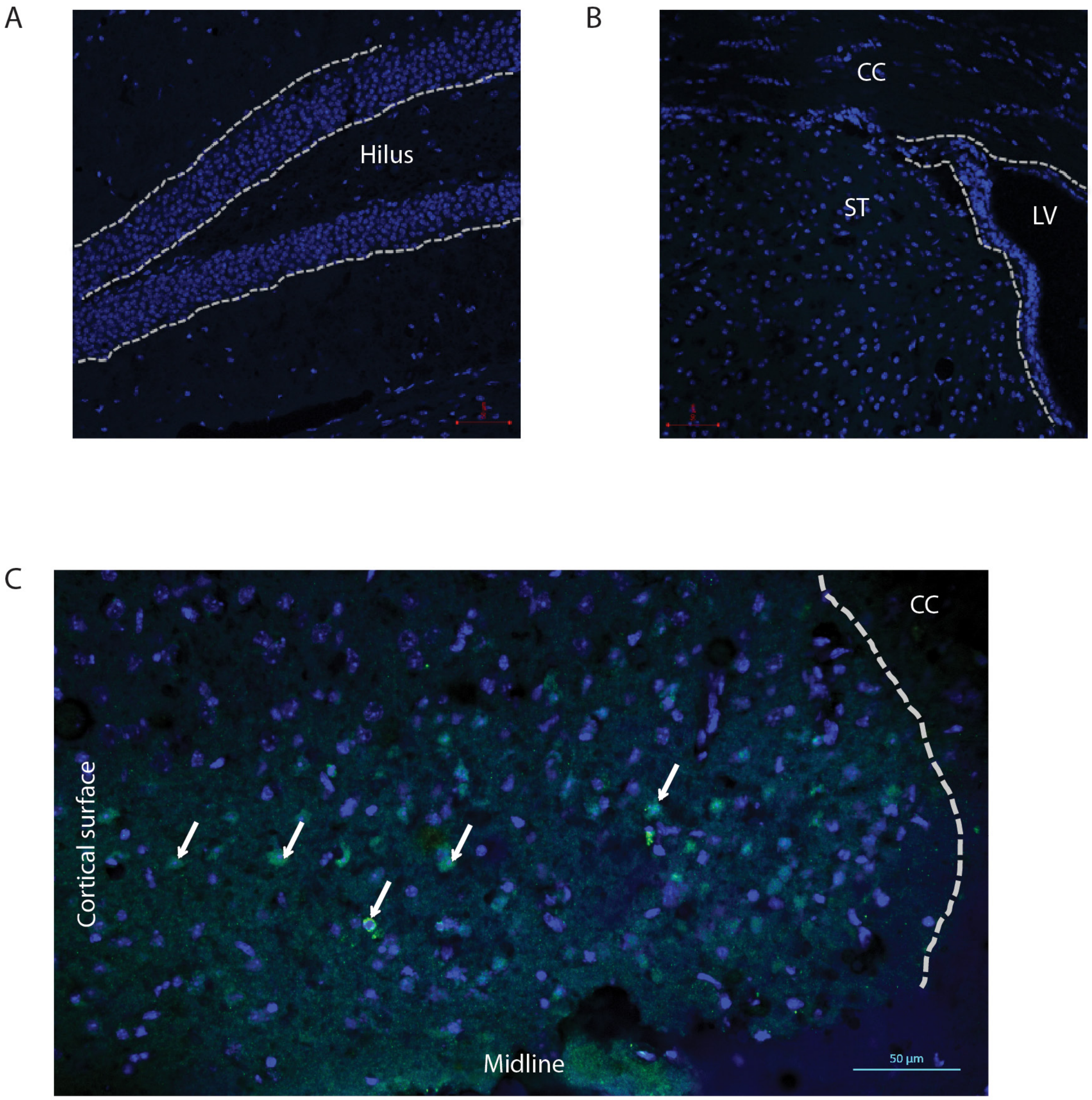

Supplementary Figure 1: The neurogenic niches in the adult mouse brain do not express Cox-2 under baseline conditions. Brain cryosections were immunostained with a Cox-2 antibody (green) and the nuclear stain hoecsht (blue). Images show the absence of Cox-2 expression in the **(A)** SVZ (dotted line) lining the wall of the lateral ventricles and **(B)** the dentate gyrus (dotted line) with the SGZ adjacent to hilus. **(C)** As a positive control, staining for Cox-2 was performed in the medial prefrontal cortex following carotid artery occlusion and stroke. SVZ = subventricular zone; SGZ = subgranular zone CC = corpus callosum; ST = striatum; LV = lateral ventricle.
